# Generation Z’s Health Information Avoidance Behavior: Insights From Focus Group Discussions

**DOI:** 10.2196/54107

**Published:** 2024-03-08

**Authors:** Chenjin Jia, Pengcheng Li

**Affiliations:** 1 School of Communication Universiti Sains Malaysia Gelugor Malaysia

**Keywords:** information avoidance, health information, Generation Z, information overload, planned risk information avoidance model

## Abstract

**Background:**

Younger generations actively use social media to access health information. However, research shows that they also avoid obtaining health information online at times when confronted with uncertainty.

**Objective:**

This study aims to examine the phenomenon of health information avoidance among Generation Z, a representative cohort of active web users in this era.

**Methods:**

Drawing on the planned risk information avoidance model, we adopted a qualitative approach to explore the factors related to information avoidance within the context of health and risk communication. The researchers recruited 38 participants aged 16 to 25 years for the focus group discussion sessions.

**Results:**

In this study, we sought to perform a deductive qualitative analysis of the focus group interview content with open, focused, and theoretical coding. Our findings support several key components of the planned risk information avoidance model while highlighting the underlying influence of cognition on emotions. Specifically, socioculturally, group identity and social norms among peers lead some to avoid health information. Cognitively, mixed levels of risk perception, conflicting values, information overload, and low credibility of information sources elicited their information avoidance behaviors. Affectively, negative emotions such as anxiety, frustration, and the desire to stay positive contributed to avoidance.

**Conclusions:**

This study has implications for understanding young users’ information avoidance behaviors in both academia and practice.

## Introduction

### Background

As digital natives, Generation Z (born between 1997 and 2012) actively uses a variety of sources for health information. However, they have limited life experience and lower cognitive ability to process large amounts of information compared with older generations [1,2]. As indicated by Robb and Shellenbarger [3], college students are able to retrieve health information on their own, but they are less confident for individual decision-making in health choices. When health risks appear, young people are often fearful and stressed, which can lead to stronger reactions such as avoiding information about the risk [1] or even withdrawing from social media [4]. The studies found that young adults, when perceiving a potential threat and lacking the ability to effectively cope, either seek health information to fulfill their information needs or avoid health information that conflicts with their cognitive beliefs or elicits unpleasant emotions [5,6].

Information avoidance refers to *any behavior designed to prevent or delay the acquisition of available but potentially unwanted information* [6]. Unlike the state of not actively seeking information, information avoidance is a deliberate act of avoiding or delaying available but unwanted information [7]. This behavior manifests in various ways, such as selective access to information from intermediary sources [8], diverting attention [9], biased interpretation of information, and selective forgetting [10]. Although information avoidance is considered the least active state of information seeking [11], these 2 behaviors are fundamentally distinct. Information seeking entails users having information needs and making efforts to fulfill those needs [12]. On the contrary, information avoidance represents a motivated decision to steer clear of information that threatens how individuals wish to think, feel, or behave [7].

Information avoidance in the health context can occur in various ways. For example, Persoskie et al [13] found that 29.4% of participants above the age of 50 years who knew they should have obtained information from their physicians still avoided seeking medical care. Similarly, a national survey in the United States showed that 31.1% of adults said they would not know their chances of developing cancer [14]. In addition, it encompasses other information-related psychological phenomena, including avoiding exposure to information that is inconsistent with one’s attitudes [15], ignoring information about a disease after it has been diagnosed [16], and other forms of defense and avoidance [17]. During a crisis, the avoidance of health information can reduce personal anxiety. However, avoiding health information may negatively impact individuals. It prevents people from digesting valuable information to improve health decisions, leading to delayed or missed disease screenings and medical visits [10].

Exploring how and why individuals seek health information has long been a focus of psychology and communication research. However, relatively little attention has been paid to the phenomenon of information avoidance, especially among specific populations. The purpose of this study is to explore how Generation Z perceives the factors that influence their information avoidance behavior, drawing on the planned risk information avoidance (PRIA) model as a theoretical guide to reveal the underlying motivations for this behavior. In the *PRIA Model*, *Sociocultural Factors*, *Cognitive Factors*, and *Affective Factors* sections, we first review the factors related to information avoidance to provide information for our qualitative investigation. We then describe the research methods and results. Finally, we review our main findings, theoretical and practical implications, limitations, and future research directions.

### PRIA Model

As information avoidance behavior has gradually gained attention from scholars, a wide range of theories and frameworks, for example, the theory of planned behavior, the stimulus-organism-response model, and the risk information seeking and processing (RISP) model, have been applied to the study of information avoidance by researchers from psychology, communication, informatics, and medical backgrounds. Some scholars have attempted to understand information avoidance from the perspective of risk communication using a cognitive model [18,19]. Of these, the RISP model is one of the most widely used models for understanding individuals’ information-seeking and -processing behaviors across a variety of environmental or health risks [20]. It assumes that the importance of perceived information insufficiency in determining the importance of proactive risk information seeking, including cognitive and psychosocial motivational drivers such as perceived hazard characteristics, affective responses, relevant channel beliefs, and subjective norms of information, which in turn influence perceived information insufficiency. Although the model has been applied to explain and predict information avoidance behavior in different crisis contexts [21-23], the shortcoming is that the correlates of information avoidance are different from information seeking, and it has been shown that the direct relationship between risk perception, insufficient information, and information avoidance has not been supported [24].

**Figure 1:**
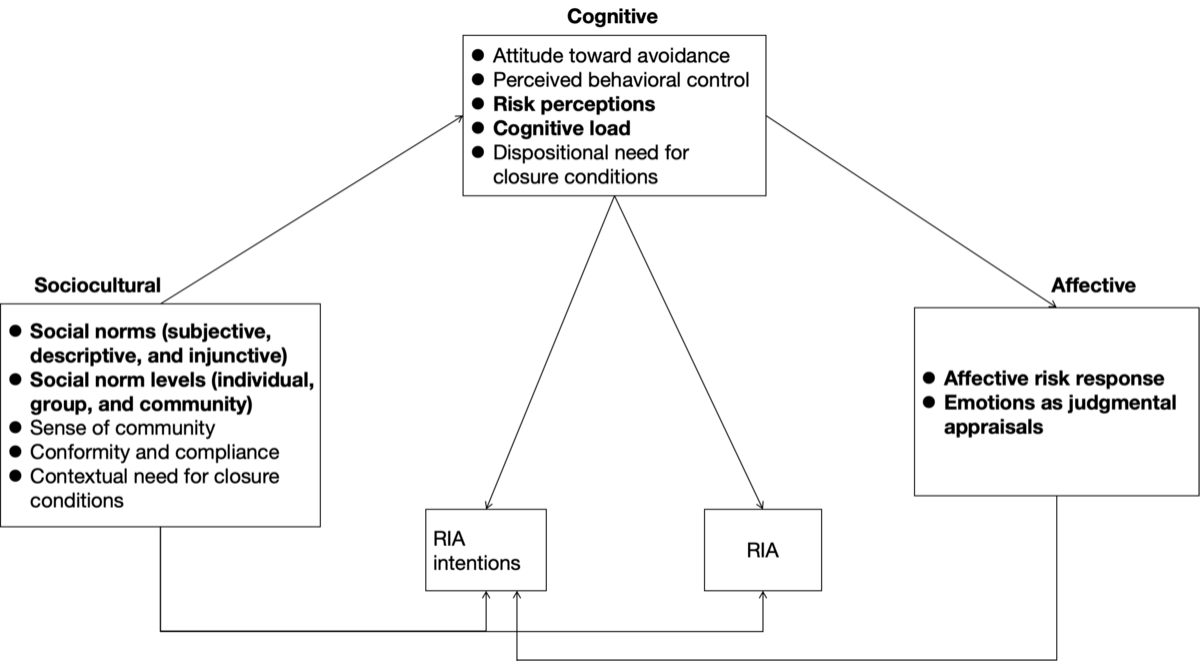
Planned risk information avoidance (PRIA) model (adapted from Deline and Kahlor). Bold text refers to the factors involved in this study.

To clarify the predictors of information avoidance behavior, Deline and Kahlor [25] proposed the PRIA model as a further complement to the extended iteration of RISP, as shown in Figure 1 [25]. They reviewed the variables in previous risk information–seeking studies and identified potential factors from the literature that may contribute to information avoidance behavior, integrating and proposing models that are more appropriate for studying information avoidance behavior. The model emphasizes the behavioral as-intended nature of risk information avoidance and explains the sociocultural, cognitive, and affective factors that underpin the behavior. Notably, Deline and Kahlor [25] pointed out that PRIA is a “pre-theoretical conceptual representation of a phenomenon...that can be used to elaborate theory, integrate research fields and test variable relationships.” Therefore, in this study, we followed the PRIA model as a theoretical framework and coding justification for exploring health information avoidance behaviors in Generation Z.

### Sociocultural Factors

The social factors encompass the investigation of how an individual’s social environment network, including the perceived pressures and expectations from loved ones and peers, influences their evaluation of risk information and subsequent behavioral responses [20]. This study focused on social norms as one of the most relevant factors guiding individual health behaviors, including health information behaviors [9,26]. Social norms can be described as “rules and standards that guide and/or constrain social behavior as understood by group members and without the force of law” [27]. The PRIA model posits a significant association between information avoidance behavior and both descriptive norms and injunctive norms [25]. This proposition is substantiated by Qu et al [28], who contend that individuals who perceive a higher degree of involvement of their social relationships in information avoidance or the approval of such behaviors are more prone to engaging in such behaviors themselves.

Indeed, health behaviors are often influenced by family, friends, and other social relationships, such as health information behaviors [23]. Link et al [9] argued that individuals would avoid health information more frequently if information avoidance was perceived to be endorsed by relevant others (injunctive avoidance norms) or was perceived to be a frequently performed behavior (descriptive avoidance norms). In contrast, Heck and Meyer [29] found that people who avoid genetic health information are evaluated more negatively and that people tend to believe that learning relevant information is the right choice.

### Cognitive Factors

Cognitive factors are related to how people perceive risk, how much they know about it, and how these factors drive information avoidance [25]. In the first place, risk perception refers to the individual’s perception of the hazardous circumstances associated with a specific risk they encounter. It is determined through subjective evaluation of the severity and susceptibility of potential risks [19,30]. Dunwoody and Griffin [31] posited that heightened risk perception may serve as a catalyst for individuals to engage in information avoidance, particularly when the information is construed as excessively menacing. However, in instances where individuals perceive a minimal likelihood of risk occurrence, they similarly exhibit an active inclination to avoid information regarding the subject matter [25].

It should be noted that risk perception may be altered by cognitive load. Cognitive load is the pressure or burden on an individual’s information-processing resources while performing a cognitive task. With limited cognitive capacity, it is difficult for people to effectively handle more than one cognitive work task at a time [32]. Information overload transpires when individuals perceive that they must process an excessive amount of information that surpasses their capacity to effectively use it, thereby resulting in an excessively burdensome cognitive load [1]. The extensive use of the internet coupled with the abundance of intricate information surpasses the information-processing capabilities of Generation Z users, frequently subjecting them to heightened levels of information overload [1]. When information overload exists, individuals strive to alleviate their cognitive load by exhibiting a proclivity for actively pursuing and embracing information that aligns with their preexisting beliefs while avoiding information that is inconsistent with prior convictions [25,33].

### Affective Factors

The avoidance of health information is an emotionally driven, maladaptive defense response [6,34]. Following the PRIA model, we focused on the valence of the emotional response (ie, positive or negative). Yang and Kahlor [35] argued that the more negative the emotional risk responses an individual perceives, the less likely they are to avoid information. Nevertheless, recent research has provided evidence that both negative and positive emotional responses can serve as motivators for information avoidance [20]. Specifically, when individuals receive health information that is associated with negative emotions (eg, getting a cancer diagnosis elicits fear) or requires them to take an unexpected behavior (eg, undergoing surgery), they choose to avoid the information [6,36]. Similarly, Dai et al [37] stated that when users feel frustrated by information overload, they may adopt emotional or behavioral coping strategies, including interrupting or avoiding information, to alleviate or prevent this unpleasant feeling.

There is no doubt that positive emotions such as hope, optimism, and excitement can also influence an individual’s behavior. Yang and Kahlor [35] posited that the more positively a person feels about risky information, the more inclined they are to avoid the information. It has been shown that information avoidance occurs when individuals have a higher degree of confidence in their own level of knowledge [22,23]. Generation Z may be inclined to avoid health information when they believe they have sufficient knowledge of it, especially if they do not find it credible or relevant to their situation [38].

## Methods

### Data Collection

We conducted 7 online focus groups between February 2023 and May 2023 consisting of 38 participants. We recruited samples using WeChat snowball sampling, online community and volunteer advertising sites, and social media advertisements (Weibo promote). Eligible participants must (1) be aged between 16 and 25 years, (2) have a high frequency of social media use (at least once a day), and (3) be experienced with health information avoidance behaviors, among other things. Before formal data collection, all participants signed a consent document agreeing that their conversations would be recorded and used for research purposes. Specifically, the written informed consent was obtained from the parents of 4 of the underage participants. The document emphasized that all records would be handled anonymously.

Data were collected using online focus groups because they may be an effective way to solicit public opinion on health and medical issues, especially from diverse and geographically dispersed participants [39]. The focus groups were conducted through Voov Meeting (Tencent Ltd), the most used online meeting platform in mainland China, which allows for instant communication and enables the moderator to coordinate the entire discussion process appropriately [40].

The first author was the moderator of the focus group discussion. The online focus groups were conducted in a semistructured discussion with participants joining in using audio. Participants were informed of the purpose of the study and introduced to the core concepts discussed (eg, What is information avoidance?). Then, the formal discussion began. The discussion was based on a practical guide to focus groups prepared by Krueger and Casey [41]. The topic guide was developed using the existing literature on attitudes and experiences of information avoidance and was tested and refined in a pilot focus group. The online focus group interview guide is presented in Multimedia Appendix 1. The group sizes ranged from 4 to 8 participants.

### Analysis

All discussions were audio recorded and transcribed verbatim with the consent of all participants. We recorded a total of 10 hours and 45 minutes of audio data from the focus group interviews. The duration of each individual interview session ranged from 75 minutes to 106 minutes, resulting in an average total length of 92 minutes per focus group. Participants’ names were replaced with P1-P38, all documents and transcripts related to the study were deidentified, and all audio files were destroyed after the transcription was complete. Theme extraction was conducted through deductive coding, whereby predefined coding categories were applied to the qualitative data based on the PRIA model. This was used by 2 trained coresearchers with extensive experience in qualitative content analysis, and they considered generational nuances as well as emerging themes that may not have been captured in the initial coding framework. Themes were extracted and coded over multiple iterations until no new themes could be generated. Coders independently coded all transcripts and discussed and compared their coding results with each other. Disagreements were resolved by checking their coding. Both coders provided coding rationalizations and jointly determined the best theme for the response. Final themes were reviewed and finalized by the coders and the first author.

### Ethical Considerations

At the time of the study, the authors' working institution had no policy of ethical clearance for internet-based studies such this one. Therefore, formal ethical approval from the institutional review board was not obtained. Nevertheless, the study was conducted in accordance with the ethical standards laid down in the 1964 Declaration of Helsinki and its later amendments or comparable ethical standards, including existing laws and regulations on personal data, privacy, and research data management. Informed consent was obtained from all participants before data collection. The collected data was strictly utilized for scientific research purposes and was not stored or used for the identification of any individual participant. At the end of the final focus group, each participant was compensated with CN ¥80 (approximately US $11) e-commerce vouchers.

## Results

### Overview

A total of 38 participants were recruited, of which 55% (21/38) were male participants. The participants were aged 16 to 25 (mean 20.9, SD 2.52) years. Moreover, 66% (25/38) of the participants were undergraduate students or had a degree. The participants had used social media for an average of 6.89 years. Detailed background characteristics of the participants are shown in Multimedia Appendix 2.

Participants agreed that health information is important, but that the information environment presented significant challenges to their understanding and processing of health information and that it was easier for them to avoid this information at times. We organized the findings in the *Irrelevant Information Topics*, *The Effects of Group Identity*, *The Conflict of Values*, *Risk Perception Discrepancy*, *Information Overload*, and *Affective Responses to Cognition* sections according to the 3 factors discussed in the literature review (sociocultural, cognitive, and affective). Multimedia Appendix 3 lists all the factors and provides citations for each subtheme.

### Irrelevant Information Topics

Participants received information and stimuli from all aspects of internet use on a daily basis. For personal information consumption, they may pay more attention to topics directly related to their daily lives, hobbies, or social relationships, and they may spend time browsing their social media circles of friends or paying attention to content related to their interests while ranking health information as secondary. This preference for the allocation of attention may cause them to ignore or avoid accessing and concerning about health information. One participant said the following:

Fashion, entertainment, and study related information takes up my free time. Health information? Nah, not really my thing...[what I’m into are] looking good, having fun, and keeping up with my academic game. Health stuff just doesn’t grab my attention, you know?

P33, aged 20 years, male participant

For social interactions, if participants consider health information to be irrelevant to their current topic of conversation, they may find it out of place and difficult to integrate with the topic or social interaction they are discussing. Many younger participants felt that there were underlying rules about the topics discussed in their social circles, which made them consciously choose to be exposed to certain information and avoid others. One participant stated the following:

Sometimes we just avoid health info because it’s not cool to talk about certain stuff. Like, Youth social circle has these unwritten rules about what’s okay to discuss and what’s off-limits.

P16, aged 18 years, male participant

For them, health information was perceived as boring and dull content, as one participant disclosed the following:

It seems like everyone agrees that health information is totally unfashionable and nothing worth talking about.

P5, aged 18 years, male participant

### The Effects of Group Identity

In the lives of young adults, the formation of their identity and integration into social groups represent a crucial developmental task. Some participants felt that if other young people in a WeChat group or other social media community preferred to avoid health risk information, then a sense of belonging as a member of the community led them to follow these avoidance norms, as one participant stated the following:

Every group has underlying norms and group rules, and since I’m a member of the WeChat group, I should follow them. It’s all about fitting in and following the group’s unspoken rules.

P18, aged 18 years, male participant

Participants with a strong sense of belonging also indicated a greater tendency to persist in the behavior if positive feedback from peers reinforced avoidance of health messages, as one participant said the following:

If all my friends think that leaving those health messages alone is what young folks should be doing, I’ll stick with it. I gotta fit in and be consistent with them.

P10, aged 21 years, female participant

Young people are often concerned about their image and identity in their social circles. They may be concerned about being seen as different or being excluded from social circles if they focus on or discuss health issues. One participant said the following:

When no one else is paying attention to health information, the fear of being judged or ridiculed by my peers is a concern, so I simply avoid it all together as well.

P34, aged 18 years, female participant

Another participant shared similar perspectives, saying the following:

I worry about being labeled and considered abnormal. So simply choose to avoid it and save us the trouble.

P38, aged 21 years, male participant

In this case, participants expressed that although individuals may be aware of the importance of health information, based on the desire for social expectations and group identity, they wanted to align themselves with others. One participant explained the following:

Honestly, I understand the significance of health info. But when I see the lack of attention or importance people generally give it in our community or society, I feel this need to conform. It’s like I wanna be part of the collective vibe.

P19, aged 22 years, male participant

### The Conflict of Values

The younger generation has easy access to a wide range of health information, but the quality and credibility of the information varies. They may face conflict over different health advice. Avoiding health information can preserve self-identity in the face of information that challenges their existing values. One participant said the following:

I totally believe in scientific health information released by official or medical professionals, while some information always promotes folk remedies and even recommends that cancer can be cured by brewing some unknown plant with water, which is unacceptable to me.

P29, aged 23 years, male participant

Some participants in this study reported that this conflict of values also manifested in the way they questioned the source of a particular piece of health information or perceived the source to be potentially biased. For example, one participant said the following:

You know that clickbait online? Those accounts don’t even publish decent health information; they are actually advertisements.

P20, aged 21 years, male participant

Another participant explained as follows:

I never read health information published by a self-media outlet, they must have published it for commercial interests. I also avoid all those accounts that promote natural remedies, energy healing, and so on, because I firmly choose modern medicine, and I will consult a doctor if I have health issues.

P27, aged 23 years, female participant

Sometimes this conflict of values comes from family members. Some participants expressed that this could make them feel helpless as older family members often share unscientific health information. One participant complained as follows:

I know my parents were concerned about our health, but they were often attracted to unscientific or deceptive health information and shared it with our family groups.

P2, aged 19 years, male participant

Younger generations perceive older generations as being poor at recognizing the credibility of health information and have a strong bias against their health literacy, with many participants expressing similar views:

Maybe it’s bias, but I avoid all health information whenever it is shared by relatives and parents.

P13, aged 21 years, female participant

As pointing out mistakes may result in family conflict, these young participants hoped to maintain a harmonious family atmosphere and chose to simply avoid health information.

In addition, a culture of self-presentation and comparison is prevalent on social media. If social media emphasizes aspects such as appearance, body shape, and quick slimming, it may cause some self-esteem damage to young users, who may feel that they are not healthy or perfect enough compared with others. To avoid conflicting with the values on social media, they chose to avoid health-related information and comparisons with others. One participant stated the following:

A lot of health information preaches perfect, healthy living, as if everyone should have a perfect body or adhere to a specific diet. In real life, I don’t stick to these norms, so sometimes I just avoid the info to keep things simpler for myself.

P17, aged 25 years, female participant

### Risk Perception Discrepancy

Risk perception refers to an individual’s ability to perceive and recognize potential risks and possible negative consequences. There were differences in the participants’ perceptions of health risks. Some participants stated that the possible consequences of health information can make them uncomfortable, especially when they see some information about serious diseases. One participant said the following:

Every time I come across health-related content, it’s like my imagination goes wild, conjuring up all these horrifying scenarios. It really gets under my skin, you know? It’s just so unsettling and makes me feel really uncomfortable.

P4, aged 22 years, female participant

Some individuals were more sensitive to the risks mentioned in the health information, which made them likely to be more concerned about health issues and potential risk factors and may have adopted more cautious and precautionary behaviors:

The health information tends to make me self-referential, and I prefer not to hear about potential health issues or underlying diseases that may apply to me. Ignorance is bliss, avoiding them keeps me from having to face possible risks and challenges head-on.

P12, aged 23 years, male participant

Health issues are often associated with negative concepts such as disease, pain, and death. As a result, young people may be more willing to focus on the positive aspects and avoid information about health risks and concerns. In contrast, some participants were less sensitive and concerned about potential risks and possible negative consequences. They may perceive themselves to be in relatively good health, with no obvious risk factors, and therefore see themselves as less susceptible to health threats. A participant declared the following:

I don’t really see health risks everywhere. When it comes to health threats, they just don’t bother me at all. I’ve got this invincible mindset, like nothing can touch me, so I don’t even care these info.

P22, aged 16 years, male participant

This perception makes individuals less interested in information about their health conditions and disease risk. In addition, there were participants who felt confident in their health knowledge or had sufficient information about the risks, and this confident judgment made them avoid the health information they thought they knew. One participant claimed the following:

I know all about those health topics, it’s just a matter of repeating that.

P8, aged 19 years, female participant

### Information Overload

The internet and social media are inundated with a plethora of health information. When individuals are exposed to an information overload that surpasses their capacity to assimilate it, information overload occurs. Most participants expressed that they were exposed to a significantly greater volume of information than what they require. One participant said the following:

As soon as I turn on my mobile phone every day, all these apps start flooding me with tons of information. There’s just too much health stuff out there, it’s like trying to drink from a fire hose.

P15, aged 23 years, female participant

The variety of information and socialization needs can be greater than young users can handle, and many participants reported being exhausted after expending too much energy to deal with these needs. One participant said the following:

It’s like a never-ending stream of articles, videos, and opinions. I’m so tired and afraid of being misinformed that I’d rather avoid them.

P33, aged 20 years, male participant

Meanwhile, participants expressed that during a health crisis they perceived a higher level of information overload, especially health-related overuse of the internet:

During COVID-19, it felt like everyone and even their grandparents were sharing health stuff left and right, you know? It didn’t matter where it came from, it was like everyone suddenly became a doctor or something. And let me tell you, it was a freakin’ info overload. True or false, all that health info was flying’ around, and I was just getting drowned in it.

P25, aged 25 years, male participant

Participants reported that at the beginning of a crisis, they would seek health information to get help because of lack of information. As more information became available, however, more than they could process, they turned to avoiding health information. One participant said the following:

I do know that it’s useful to know about health information, but it takes more effort to always check out that information and to assess the quality of the content of the information. I’m already swamped with work and don’t have the time and energy to delve into these things.

P24, aged 21 years, female participant

### Affective Responses to Cognition

Cognitive factors relate to participants’ understanding and processing of information, whereas affective factors relate to participants’ emotional responses and emotional experiences with health information. For participants, different cognitive assessments may lead to different affective responses. For example, the abundance of diverse health information from various sources could lead individuals to experience an influx of different perspectives. Under such circumstances, some participants reported that they may encounter confusion, contradiction, and a sense of being lost in navigating health information:

It’s like conflicting info everywhere, and we end up feeling confused and unsure about what to believe. Sometimes I just avoid it altogether to save my sanity.

P26, aged 20 years, male participant

Furthermore, participants felt that the excessive cognitive load owing to information overload sometimes caused them to feel overly stressed and anxious, and to minimize emotional tension, participants intentionally stayed away from stressful situations. One participant said the following:

Sometimes it feels like you gotta absorb every piece of info to stay healthy, but it’s impossible. It’s like this constant fear of missing out or not doing enough, and it can give you major anxiety.

P28, aged 21 years, male participant

Sometimes participants may show stronger emotional response when confronted with information about health risks. Participants with higher risk perceptions said that the possible consequences of the risk could cause them fear, especially when they saw some information about serious diseases:

Those anti-smoking ads on cigarette packs are always there...[those pictures] just make me cringe. I mean, I do smoke once in a while, but my best friends smoke like a chimney. So, to save myself from feeling grossed out and uncomfortable, I just steer clear of any health info about lungs.

P7, aged 25 years, female participant

When faced with discomforting or anxiety- and fear-inducing health information, people tend to adopt emotional defense mechanisms to avoid negative emotions. Participants indicated that their avoidance of health information reduces emotional upset and stress. One participant said the following:

Diving into a bunch of health info just makes me go off on a tangent. I’m all about staying positive and living a healthy life, you know? Happiness is what matters most, right? So, no need to get hung up on all that health stuff.

P35, aged 23 years, male participant

## Discussion

### Principal Findings

The results of this qualitative study shed light on how Generation Z perceives the social, cognitive, and affective factors in their health information avoidance and provides insights for understanding and addressing these behaviors. Two main types of health information avoidance behaviors were observed among participants, regardless of education or age: passive and active avoidance. Passive avoidance occurs mainly under social influence, whereas active avoidance is a stress-coping mechanism used to temporarily avoid confusion when exposed to excessive information. Our findings also showed that the level of risk perception had an inverted U-shaped relationship with information avoidance in participants, with both high- and low-risk perception leading to avoidance. When faced with excessive or complex information, Generation Z experiences increased cognitive load and psychological discomfort, such as stress and anxiety, pushing them to avoid information. In the *Comparison With Prior Work* section, we consider the implications of these findings in relation to existing literature and discuss their significance.

### Comparison With Prior Work

This study provides an insightful perspective on the health information behaviors of the younger generation. This indicates that health information avoidance behaviors should not be viewed as an isolated way of information processing but rather as part of the development of healthy information behaviors among young internet users.

Specifically, our findings are consistent with previous research showing that social norms continue to provide strong socially and culturally negotiated, contextually relevant, group identity-based behavioral norms in Generation Z socialization [9]. Young generations commonly select and adapt their information behaviors based on the behavior and acceptance of their social relationships [28]. This study further points to the finding that, as group interaction is one of the mechanisms through which norms emerge and develop, when peers are not interested in health information, they may be concerned that they will be seen as different or excluded from their social circle if they focus on or discuss health issues. Thus, even if at the individual level they believe that they should be concerned about health information, they may perceive that the dominant norms of the community do not value health information. At this point, health information is no longer cognitively processed, resulting in a passive, involuntary behavior. This supports previous research [25], where subjective avoidance norms cause someone to believe that their friends expect relevant health information possibilities to be avoided in conversations, forcing them to change their personal level norms to be more in line with what they perceive as social norms. Meanwhile, we found no mention in the previous literature that social media widely disseminates information about health views such as the perfect body and particular diets, which may have an influence on young people’s self-identity and behavior. When participants realize that they are not aligned with these social norms, they may choose to avoid this information to minimize conflict or discomfort with social norms.

Earlier studies indicated that risk perception is negatively related to information avoidance [22]. In this study, we found that the level of risk perception of the younger generation was more differentiated from their information avoidance behavior, with both high- and low-risk perceptions leading to information avoidance. Therefore, we suggest that there is an inverted U-shaped relationship between the level of risk perception and Generation Z’s health information avoidance behavior, which is consistent with the proposition of Deline and Kahlor [25]. Moreover, older participants had higher levels of risk perception, whereas younger participants generally showed lower risk perceptions. This difference may be because higher risk perceptions motivate individuals to avoid information if they are perceived as threatening [31]. As mentioned by Narayan et al [42], active avoidance is a short-term rejection of information and is more of a stress-coping mechanism to temporarily avoid confusion to cope with the risk information. Faced with possible health risks, some young people may experience concerns, and as a defense mechanism, they may choose to avoid information related to health risks to reduce feelings of unease. However, other younger people may lack sufficient knowledge and experience regarding health risks, leading to a diminished sensitivity in risk perception. They are more inclined to prioritize immediate pleasures and adventures, potentially resulting in insufficient attention being given to long-term health risks. In addition, they may exhibit a certain degree of optimistic bias, wherein they hold excessively optimistic views regarding the risks of negative events befalling themselves. They may perceive a lower risk of developing severe illnesses and consequently engage in information avoidance to maintain a lower perceived threat level.

In addition, we found that both the quantity and quality of information can increase the cognitive load of Generation Z, pushing them to avoid information. On the one hand, in line with previous studies, the information overload perceived by the young people in this study made it difficult for them to process and use information effectively [24,43]. When millennials are faced with a plethora of information and the pressure to filter it, there may be a stronger tendency to avoid information that is perceived as less important or irrelevant. On the other hand, our findings show that young people, when faced with information and sources prone to value conflicts, tended to avoid health information to avoid value and family relationship conflicts. This supports a previous study [33], as people often use heuristic decision-making and information filtering strategies during information processing, young people tend to assess the credibility of health information on the basis of their prior knowledge and avoid all information from sources that they perceive as low credibility. Both nonprofessional and nonofficial media were identified as low credibility sources, including information sharing from older family members.

Finally, we found that whether during a health crisis or daily health information dissemination, Generation Z experiences psychological discomfort and negative affective stress, such as stress, anxiety, and frustration, in situations of high cognitive load. Song et al [44] found that perceived information overload significantly predicted the level of anxiety in people’s information behavior and positively influenced health information avoidance behavior. Moreover, when young people need to control their fear of risk, they may deny the existence of a health threat by avoiding health information as a way to reduce anxiety or remain hopeful and optimistic.

### Implications

This study provides several important implications. To the best of our knowledge, this is one of the first studies guided by the PRIA model to explore Generation Z’s health information avoidance behavior. Most studies have focused on Generation Z’s seeking of health information rather than their avoidance behavior, for example, Basch et al [45] and Zhou and Roberto [46]. Our study provides new insights into the health information–processing behaviors of the Generation Z. More precisely, it breaks through the stereotype of Generation Z as digital natives in complete control of social media, whereas sometimes cognitive and emotional stresses cause them to avoid information that could cause psychological distress or to stay away from social media temporarily [1]. We explored the relationship between social norms, cognitive load, emotion, and health information avoidance behaviors from the perspective of Generation Z, providing empirical support for the propositions of Deline and Kahlor [25], which complement existing research in communication psychology. As one of the few qualitative studies examining information avoidance behavior, our study has potential value beyond its contextual limitations. Future research could be generalized to other generations, or to information avoidance behavior in one specific disease.

Furthermore, our study has practical implications for social media users and platforms. Short-term avoidance of health information may help reduce feelings of being overwhelmed owing to high cognitive stress and information overload. In the long term, avoiding health information may have adverse effects on health and well-being [16]. For Generation Z users, we suggest engaging in self-monitoring and self-assessing their psychological state related to health information, thereby promoting healthy information use habits and maintaining psychological well-being. For social media platforms, we offered new insights into the underlying reasons behind Generation Z users’ avoidance of health information. It is crucial for platforms to provide personalized services to Generation Z users by developing content filtering and credibility feedback functions that cater to their information consumption needs and preferences.

Finally, for health education development, it is important to improve health literacy among all health system stakeholders so that they are able to filter the required information [21]. Information overload is a serious barrier to health literacy, reducing people’s understanding of health information and limiting their ability to evaluate and act on it [47]. Therefore, we recommend that health literacy training be part of the health education program for university students, whereas for young employees, regular health seminars can be arranged to improve health literacy. Exposure to high volumes of health information causes cognitive and emotional stress in young people, and they should be provided with normalization training and guidance during health crises to help them better process health information.

### Conclusions

This study explores the negative side of the use of health information in Generation Z and provides empirical evidence on the negative processing of risk information among young people. Using the PRIA model, this study revealed how young people perceive the factors that influence their avoidance of health information. We found that social norms can influence Generation Z’s health information behavior through normative pressure and group influence. Moreover, there is an inverted U-shaped relationship between risk perception and the information avoidance behavior of Generation Z, which means that either high- or low-risk perception could lead to information avoidance. Finally, information overload and low credibility increase the cognitive load of young people, causing them to experience negative emotions and therefore choose to avoid information to relieve stress.

However, this study has some limitations. We mainly focused on participants’ perceptions and experiences of health information avoidance, rather than directly observing their actual behaviors. Although perceptions provide valuable insights, they may not always align with participants’ realistic behaviors. Future research could incorporate behavioral measures to supplement self-reported data to provide a more comprehensive understanding of health information avoidance behaviors. In addition, rapid advances in technology and information environments may affect changes in health information behaviors. Given the dynamic nature of Generation Z’s information consumption behaviors, future research could expand this study to explore other health information behavioral similarities or differences in underserved populations.

## Appendix

Multimedia Appendix 1 Initial sample semistructured focus group guideline.

Multimedia Appendix 2 Focus group participants’ background characteristics.

Multimedia Appendix 3 Coding scheme for participants’ health information avoidance behavior.

## Abbreviations

PRIA: planned risk information avoidance
RISP: risk information seeking and processing
