# Supplementary material for: Generation Z’s Health Information Avoidance Behavior: Insights From Focus Group Discussions
Source: J Med Internet Res. 2024 Mar 8;26:e54107. doi: 10.2196/54107 (PMC10960220; doi:10.2196/54107)
Supplement: Multimedia Appendix 1 [file jmir_v26i1e54107_app1.docx]

Multimedia Appendix 1. Initial Sample Semi-Structured Focus Group Guideline

## Introduction (5 mins)

The researcher performs a self-introduction, goes over the consent information with the participant, introduces the whole interview schedule, and emphasizes again: “*At any point, for any reason, you are welcome to take a break or stop the interview altogether*.”

## Concept explanation or confirmation (5 mins)

The researcher re-explains or answers questions about the core concepts involved in this research: health information and health information avoidance.

## Opening Questions (Approximately 60 mins)

**Initial questions**

- Are you consciously avoiding exposure to certain health information in your daily life? Could you provide specific examples?

**Some possible questions**

- What factors do you consider when deciding whether to engage with a health topic?
- How do you perceive the influence of sociocultural factors, such as social norms and values, on your behavior of avoiding specific health information?
- What role does social pressure, particularly from peers or social media, play in your behavior of avoiding health information?
- Do you feel pressured to conform to certain norms or expectations?
- Can you give an example of when social pressure impacted your health decisions?
- How has information overload affected your acquisition and processing of health information?
- How do you cope with the abundance of health information from various sources?
- To what extent does cognitive load contribute to your avoidance of specific health information?
- Do you find yourself confused or conflicted by contradictory health information?
- How do affective factors influence your behavior of avoiding health information?
- Do concerns or fears lead you to deliberately avoid certain health information?
- In your opinion, what impact does the behavior of avoiding health information have on your overall health and well-being? Please share any personal experiences or perspectives you may have.
- Are there specific topics or subjects that make you more inclined to avoid related health information? Could you provide examples to illustrate this tendency?

**Concluding questions**

- Is there anything else you want to tell us?
- Do you have anything to ask me?

## Background survey (5 mins)

The researcher asks the participant to fill in the background questionnaire to learn his/her population and social characteristics and answers questions that the participant may encounter.

## Conclude the interview (5 mins)

The researcher thanks the participants and concludes the online focus group interview.
