# Supplementary material for: Generation Z’s Health Information Avoidance Behavior: Insights From Focus Group Discussions
Source: J Med Internet Res. 2024 Mar 8;26:e54107. doi: 10.2196/54107 (PMC10960220; doi:10.2196/54107)
Supplement: Multimedia Appendix 2 [file jmir_v26i1e54107_app2.docx]

Multimedia Appendix 2. Focus group participants’ background characteristics.

| **ID** | **Gender** | **Age** | **Education background** | **Social media usage (years)** |
| --- | --- | --- | --- | --- |
| P1 | F | 17 | High school | 4 |
| P2 | M | 19 | Undergraduate | 5 |
| P3 | M | 20 | College diploma | 6 |
| P4 | F | 22 | Bachelor’s degree | 8 |
| P5 | M | 18 | Undergraduate | 7 |
| P6 | F | 24 | Postgraduate | 10 |
| P7 | F | 25 | College diploma | 8 |
| P8 | F | 19 | Undergraduate | 7 |
| P9 | M | 22 | Postgraduate | 6 |
| P10 | F | 21 | Undergraduate | 8 |
| P11 | F | 17 | Undergraduate | 4 |
| P12 | M | 23 | Postgraduate | 7 |
| P13 | F | 21 | Undergraduate | 6 |
| P14 | M | 22 | Bachelor’s degree | 7 |
| P15 | F | 23 | College diploma | 6 |
| P16 | M | 18 | Undergraduate | 6 |
| P17 | F | 25 | Bachelor’s degree | 8 |
| P18 | M | 18 | Undergraduate | 8 |
| P19 | M | 22 | Bachelor’s degree | 10 |
| P20 | M | 21 | Undergraduate | 6 |
| P21 | F | 24 | College diploma | 12 |
| P22 | M | 16 | High school | 4 |
| P23 | F | 18 | Undergraduate | 5 |
| P24 | F | 21 | Undergraduate | 8 |
| P25 | F | 25 | Bachelor’s degree | 11 |
| P26 | M | 20 | Undergraduate | 4 |
| P27 | F | 23 | Postgraduate | 8 |
| P28 | M | 21 | Bachelor’s degree | 5 |
| P29 | M | 23 | Bachelor’s degree | 10 |
| P30 | M | 21 | Bachelor’s degree | 5 |
| P31 | F | 17 | High school | 4 |
| P32 | M | 23 | Postgraduate | 10 |
| P33 | M | 20 | Undergraduate | 7 |
| P34 | F | 18 | High school | 4 |
| P35 | M | 23 | Bachelor’s degree | 10 |
| P36 | M | 24 | Bachelor’s degree | 7 |
| P37 | M | 19 | Undergraduate | 4 |
| P38 | M | 21 | Undergraduate | 7 |
