# Supplementary material for: Generation Z’s Health Information Avoidance Behavior: Insights From Focus Group Discussions
Source: J Med Internet Res. 2024 Mar 8;26:e54107. doi: 10.2196/54107 (PMC10960220; doi:10.2196/54107)
Supplement: Multimedia Appendix 3 [file jmir_v26i1e54107_app3.docx]

Multimedia Appendix 3. Coding scheme for participants’ health information avoidance behavior

|  | **Subthemes** | **Definitions** | **Example quotations** |
| --- | --- | --- | --- |
| Sociocultural factors | Irrelevant Information Topics | Irrelevant information topics refer to subjects or discussions that are not directly related to the main topic or focus of a conversation, context, or situation. | All that wellness and chronic disease information that older people are so keenly interested in. It’s got nothing to do with us young folks. (P37, 19 years, male) |
|  | The Effects of Group Identity | The effects of group identity refer to the impact that one’s identification with a particular social group has on their thoughts, attitudes, behaviors, and sense of self. | When no one else is paying attention to health information, the fear of being judged or ridiculed by my peers is a concern, so I simply avoid it all together as well. (P34, 18 years, female) |
| Cognitive factors | The Conflict of Values | The conflict of values refers to a situation where individuals or groups hold different or opposing sets of values or beliefs. | The internet is full of unscientific health information that is totally misleading, just don't view any of it! (P6, 24 years, female) |
|  | Risk Perception Discrepancy | Risk perception is an individual’s ability to perceive and recognize potential risks and possible negative consequences. | **High risk perception:** If I dive into more health information, I might become overly worried. I’m afraid of discovering that I might have some illness. (P10, 21 years, female) **Low risk perception:** I don’t really see health risks everywhere. When it comes to health threats, they just don’t bother me at all. I’ve got this invincible mindset, like nothing can touch me, so I don’t even care these info. (P22, 16 years, male) |
|  | Information Overload | Information overload refers to a state in which the amount of information exceeds an individual’s information processing capacity. | There’s so much health info out there. It’s like a never-ending stream of articles, videos, and opinions. We don’t know who to trust, so sometimes we just avoid it all. (P28, 21 years, male) |
| Affective factors | Confusion | Confusion refers to a state of being overwhelmed or uncertain. | Just to prevent the flu, this information claims that Chinese medicine is useful, and other information declares that Chinese medicine is useless, which is so confusing that I just don't browse at all. (P14, 22 years, male) |
|  | Anxiety | Anxiety refers to uneasiness in response to stress or perceived threat or worry about uncertainty. | Sometimes it feels like you gotta absorb every piece of info to stay healthy, but it’s impossible. It’s like this constant tension of missing out or not doing enough, and it can give you major anxiety. (P28, 21 years, male) |
|  | Fear | Fear refers to a strong negative emotion, usually associated with a threatening, dangerous, or scary situation, which causes feelings of nervousness, dread, or panic. | When I see health information related to cancer, I am especially prone to associate it with bad consequences of the disease, and the more I think about it, the more fearful I become, and it’s easier for me to avoid that information. (P4, 22 years, female) |
